# A peptide-based medical device for the selective removal of inflammatory factors from the blood of people with sepsis

**DOI:** 10.1038/s43856-025-01187-w

**Published:** 2025-10-27

**Authors:** Giovanni Cappello, Laura Cresti, Clelia Cortese, Letizia Bocchi, Elena Bianchini, Filippo Carlucci, Marcello Fiorini, Jlenia Brunetti, Chiara Falciani, Luisa Bracci, Alessandro Pini

**Affiliations:** 1 Department of Medical Biotechnology, University of Siena, Siena, Italy; 2 Medica S.p.A., Medolla (MO), Italy; 3 Clinical Pathology Unit, Azienda Ospedaliera Universitaria Senese, Siena, Italy; 4 SetLance srl, Siena, Italy

**Keywords:** Medical research, Diseases, Antimicrobials

## Abstract

**Background:**

Sepsis is a complex life-threatening clinical condition associated with significant morbidity and mortality. It is usually triggered by an infection, the cellular response to which progresses, involving the innate immune system and leading to a cytokine storm that can provoke death.

**Methods:**

The device is based on the peptide SET-M33, an antimicrobial molecule with strong antibacterial activity. The peptide was conjugated with a biocompatible agarose matrix via a covalent sulfonic bridge, then encapsulated in a device to insert in a circulation system. Here we describe a medical device that selectively and simultaneously remove the major triggers of the onset and progression of sepsis from the blood of sepsis patients. These include live bacteria and their components, such as lipopolysaccharides (LPS) and lipoteichoic acids (LTA), as well as C reactive protein (CRP).

**Results:**

In a human serum assay, the device removes 85% of LPS, >80% of LTA, >99% of live Gram-positive and Gram-negative bacteria in serum, and >94% of CRP. In ex vivo animal model in which murine blood spiked with known amounts of LPS, the device removes >80% of the endotoxin. After circulation in the device, the serum shows no significant change in protein content, this confirms that the device does not change the molecular profile of the blood.

**Conclusions:**

The instrument here described is a prototype with strong potential for clinical applications.

## Plain language summary

Sepsis occurs when a person’s immune system overreacts to an infection. Components within the blood can make sepsis worse. We made a medical device to remove components within the blood that make sepsis worse from people with sepsis. We show it is stable at room temperature and can work on human blood and in a mouse model. Further development may enable it to be used to treat people with sepsis.

## Abstract (web summary)

Cappello et al. describe an innovative medical device, using the antimicrobial peptide SET-M33, for the treatment of people with sepsis. This device removes live bacteria, endotoxins and C-reactive protein from the serum of sepsis patients.

## Introduction

The Third International Consensus (Sepsis-3) defined sepsis as “organ dysfunction caused by a dysregulated host response to infection”, for the first time underlining the crucial role of the innate and adaptive immune responses in the development of the clinical syndrome^1,2^. The Global Burden of Disease studies for the period 1990–2017 estimated 48.9 million cases in 2017 with 11 million deaths, while emphasizing an overall decrease in incidence (37% decrease) and mortality (52.8% decrease) compared to 1990^3^. However, the situation remains of particular concern.

After the initial episode of sepsis, an increased risk of mortality persists for more than 2 years after hospital admission, and may be associated with secondary infections and vascular changes^4^. In patients with sepsis, an inflammatory response loses its localized nature and becomes systemic with further errors in regulation, and as it progresses, it is characterized by fever, increased respiratory rate and other metabolic, hormonal and coagulative alterations, eventually leading to extremely low blood pressure^1,5,6^. These are due to upregulation of pro- and anti-inflammatory pathways that lead to system-wide release of cytokines, mediators and pathogen-related molecules, resulting in activation of coagulation and complement cascades.

Early diagnosis of sepsis is crucial for a better and faster clinical outcome. Lactic acid, procalcitonin (PCT) and sequential organ failure assessment (SOFA) score are useful biomarkers to assess the severity of sepsis, predict septic shock and reach an early diagnosis^7–10^. Many studies have reported elderly subjects with elevated SOFA scores and lactic acid concentrations among deceased patients^11,12^.

Today, standard therapy for sepsis consists mainly of attempting to eliminate the focus and timely administration of empirically targeted antibiotics (causal therapy). Additional intensive care measures are also used for individual organ support, like vasopressor administration, mechanical ventilation and renal replacement therapy (supportive therapy). Although outcomes are improving, there is no approved pharmacological treatment specific to sepsis, although it is widely accepted that early intervention is crucial for success. Live bacteria and bacterial endotoxins are simultaneously in circulation in sepsis patients^13–17^. Since bacterial infections are the major triggers of the onset of sepsis, prompt antibiotic therapy is reported to be the only effective therapeutic procedure^18^. However, misuse of antibiotics can cause dead bacteria to release excess bacterial toxins, thus furthering progression. The lack of specific treatments has promoted research mainly focused on attenuating the hyper-inflammatory state and microcirculatory disturbances associated with sepsis.

Recognition and removal of pathogen-derived molecular patterns (PAMPs, e.g., endo- and exotoxins, lipids and DNA sequences) or endogenous host-derived danger signals (damage-associated molecular patterns, DAMPs) are thought to be important strategies for abating so-called ‘immunoparalysis’^19,20^.

The severity of sepsis is partly determined by the pathogen responsible for the initial infection. Sepsis was originally thought to result mainly from Gram-negative pathogens. Current epidemiology now suggests a bigger role of Gram-positive pathogens^21,22^. Lipopolysaccharide (LPS, endotoxin), a product of the outer Gram-negative bacterial wall, is the principal bacterial toxin to induce an immune response involving the release of cytokines and other mediators^13–16^ and causing vasodilation, endothelial leakage and organ dysfunction^23–25^. Lipoteichoic acids (LTA) from Gram-positive bacteria are often involved in the evolution of sepsis^17^. LPS and LTA are the main bacterial PAMPs to trigger expression of the above pro-inflammatory mediators in host cells.

Different medical devices to purify the blood of sepsis patients are in use in intensive care units (ICUs). They remove sepsis-associated mediators or endotoxins by hemofiltration or plasmapheresis^26,27^. Oxiris®, Toraymyxin®, CytoSorb®, Seraph100®, Alteco LPS Adsorber®, HA330® are popular examples. Oxiris is a hollow fiber acrylonitrile and methalylsulfonate (AN69) membrane that removes large molecular-weight molecules by membrane sieving^28^. Toraymyxin is a device containing polystyrene-derived woven fibers with the antibiotic polymyxin B immobilized on the surface. It is used for selectively removing endotoxins from the circulation of patients^29^. CytoSorb is a device containing polymer beads that adsorb cytokines non-specifically^30–32^. Seraph uses covalently immobilized heparin for non-specific removal of mediators involved in sepsis. Alteco LPS Adsorber exploits a matrix derivatized with a synthetic peptide that selectively binds endotoxin from Gram-negative bacteria in the bloodstream^33^. HA330 is a hemoperfusion device with electrical and porous resin for the removal of cytokines and perhaps endotoxins. Few other devices are being developed for the removal of PAMPs, cytokines or immune cells^34^.

None of the commercially available devices or those being developed are reported to selectively and simultaneously remove LPS, LTA, live bacteria and possibly inflammatory factors. Thus, these devices produce non-uniform and too often ineffective clinical outcomes^20,35^. Many studies of such devices were terminated prematurely because they failed to improve 28-day mortality, vasopressor requirements, ventilation days and length of stay in ICU with respect to conventional membranes^36,37^.

The peptide SET-M33 is a polymeric molecule reported to have strong antibacterial activity against infections by major multi-resistant pathogens^38–40^. Its safety has already been tested in animals^41^. Its mechanism of action is based on specific binding to the bacterial surface by targeting negative-charged molecules such as certain proteins, LPS, LTA and anionic phospholipids. After binding, the amphipathic structure of the molecule disrupts the bacterial membrane, causing bacterial death^42^.

Here, we describe a different application of the peptide, which exploits SET-M33’s mechanism of action to bind live or disrupted bacteria and bacterial toxins (LPS and LTA) in order to selectively remove these major triggers of sepsis in ICU patients at the onset or during the progression of sepsis. This device also simultaneously reduces endogenous inflammatory factors such as CRP in the serum of sepsis patients.

The prototype is, as far as we are aware, the first medical device described in the literature that can simultaneously remove live bacteria and bacterial toxins, sharply reducing inflammatory parameters in the serum of sepsis patients. The prototype consists of a cartridge containing agarose resin conjugated with tetrameric SET-M33, designed to be inserted in an extracorporeal circulation system.

The device was tested with human serum and proved capable of removing 85% of LPS, >80% of LTA, >99% of live Gram-positive and Gram-negative bacteria spiked in serum, and >94% of CRP. In an ex vivo animal model where murine blood was processed after the animal was challenged with known amounts of LPS, the device proved able to remove >80% of endotoxin. After circulation in the device, serum did not show any significant change in protein content, confirming that passage in the device did not modify the molecular profile of blood.

## Methods

### Peptide production

SET-M33-cys peptide in tetrabranched form was solid-phase synthesized with an automated Syro multiple-peptide synthesizer (MultiSynTech, Witten, Germany), using 9-fluorenylmethoxycarbonyl (Fmoc) chemistry and activation of the carboxylic group with 2-(1H-benzotriazole-1-yl)−1,1,3,3-tetramethylaminium hexafluorophosphate/N-ethyldiisopropylamine. After amino-acid coupling, the Fmoc group was removed with piperidine 40%/dimethylformamide solution. TentaGel® S RAM resin (Rapp Polymere GmbH), a polymer-insoluble solid support (poly(oxyethylene)−2,4-dimethoxybenzhydrylamine), was used for the synthesis. Side-chain-protecting groups were tert-butoxycarbonyl (Boc) for Lys, 2,2,4,6,7-pentamethyldihydrobenzofuran-5-sulfonyl for Arg, tert-butyl for Ser and triphenylmethyl group (Trt) for Cys. The first amino acid coupled to the resin was Fmoc-NH-Cys(Trt)–COOH. Fmoc-NH-PEG(4)–CH_2_–CH_2_–COOH was added in the second step, and Fmoc-Lys(Fmoc)–OH was used twice to build the lysine core, followed by nine sequential additions of Fmoc amino acids to complete the tetrabranched peptide KKIRVRLSA. The product was then cleaved from the resin, deprotected with trifluoroacetic acid (TFA) containing triisopropylsilane, water and 1,2-ethanedithiol (EDT), and precipitated with diethyl ether.

Finally, the peptide was frozen overnight at −20 °C in a 5% aqueous solution of acetic acid and then freeze-dried for 48 h in a ScanVac freeze dryer. The crude peptide was purified by high-performance liquid chromatography (HPLC) using a reversed-phase C18 (octadecylsilane) column (XBridge peptide BEH C18 Waters, 300 Å, 10 µm, 19 × 250 mm) with 0.1% TFA/water as eluent A and acetonitrile as eluent B, performing a linear gradient from 83:17 A/B to 70:30 A/B in 40 min.

The identity and purity of the peptide were finally confirmed by reversed-phase chromatography on a Phenomenex C18 analytical column and by MALDI-TOF mass spectrometry. Mass spectrometer characterization (Ultraflex tof utx-00699 (Bruker Daltonix) was done in positive-voltage linear mode.

The monomer, dimer and tetramer synthesis of SET-M33 have only minor differences: the tetrameric structure needs a core of three lysines, whereas the dimeric structure needs only one lysine core^43^.

### SET-M33-cys conjugation with biocompatible matrix

SET-M33-Cys peptide was conjugated with agarose beads (SulfoLink™ Coupling Resin) in two rounds. The SulfoLink Coupling Resin is derivatized to contain iodoacetic groups that react specifically with free sulfhydryl at pH 7.5–9.0, present in the peptide. The 12-atom spacer arm minimizes steric hindrance, ensuring efficient binding interactions.

After removing the storage solution from the SulfoLink resin, the peptide was dissolved to 1 mg/mL in coupling buffer (50 mM Tris, 5 mM EDTA-Na; pH 8.5). The two rounds of conjugation were as follows. The peptide was incubated with the beads for 45 min. The binding rate of the peptide to beads was assessed by measuring the peptide present in the *flow*-*through* of the two immobilization cycles. Measurement was made by HPLC, comparing the area of the peptide peak before and after immobilization. Nonspecific sites of the agarose beads were saturated with 50 mM L-cysteine–HCl solution in coupling buffer. After a 45 min incubation, the agarose beads with the immobilized peptide were equilibrated with PBS (0.05% sodium azide). The bond between the peptide and agarose beads is very stable and long-lasting.

### Quantification of LPS

Quantification of LPS was obtained using the Pierce Chromogenic Endotoxin Quant Kit (Thermo Fisher Scientific) following standard procedures suggested by the provider. It is a quantitative and highly sensitive assay using amebocyte lysate derived from Limulus blood (LAL) for the quantification of bacterial endotoxin.

To perform the test, a standard curve was prepared by diluting the endotoxin stock of *E. coli* Serotype O111:K58(B4) (0.1–1.0 EU/mL) and then used for sample measurement.

### Quantification of LTA

The LTA content of a sample was quantified using a commercial LTA ELISA kit (MyBioSource). A standard curve (7.8-500 pg/mL) was prepared using a lipoteichoic acid from *S. aureus* (control standard), and then standard procedures described by the provider were used for the test.

### Bacterial colony count

Gram-negative (*E. coli* ATCC 33780™) and Gram-positive (*S. aureus* USA300)^44,45^ bacteria were used. Serum containing bacteria was obtained as follows. A pre-culture of bacteria was prepared in appropriate medium (LB for *E. coli* and TSB for *S. aureus*).

After two dilutions (1:25 and 1:20) in decomplemented serum (2 h at 56 °C), the sample was incubated in contact with the device at room temperature under slow rotation (2 h).

To quantify the bacterial removal capacity of the device, samples collected after said incubation were plated in agar medium. The CFUs growing were measured in input and output samples. Data was processed using GraphPad Prism 6 software.

### Quantification of CRP

To evaluate removal of CRP, samples were prepared by reconstituting  human C-reactive protein (Hu CRP) in their appropriate ELISA test buffers according to the protocol directions. A cartridge loaded with non-conjugated resin was used as negative control. Samples were incubated under constant rocking for 2 hours with the resin. Hu CRP was quantified by a commercial Hu CRP ELISA kit (Invitrogen) following standard procedures suggested by the provider. A standard curve (18.75–1200 pg/mL) was prepared using Hu CRP serially diluted in standard diluent buffer.

### Stability test

Devices containing SET-M33 in tetrameric form were stored at room temperature and 4 °C and tested at time zero and after 30 days. In the removal test, after two hours of incubation, serum containing *E. coli* ATCC 33780 was withdrawn from the device, diluted and plated in LB agar. This procedure was carried out twice. Devices without conjugated peptides were used as controls in both sessions.

### Blood chemistry measurement

Pools of serum from healthy subjects and sepsis patients were tested. The blood was provided in the form of a pool of anonymous sera by the Clinical Pathology Laboratory of the Siena Hospital. The blood pool used in the experiments is completely anonymous; no information on the origin of individual donors is available for tracking. No informed consent or request for Institutional Review Board (IRB) approval was required because all samples were totally anonymous and intended for disposal. IRB approval was not required for the non-animal-based parts of the study. This procedure was performed in accordance with Regulation (EU) 2016/679 (GDPR) and the provisions of the Italian Data Protection Authority regarding the use of anonymous data and biological material for research purposes.

Before the evaluation of clinical parameters, the sera were circulated in contact with devices at room temperature for 2 h. Samples were then collected and analyzed. The input serum was also analyzed as a control.

The percentage of serum proteins in the samples was analyzed by capillary electrophoresis (Capillarys 2 Flex Piercing—Sebia). The following parameters were measured: C-reactive protein, total proteins, albumin (analyzer series Roche Cobas c702 module) and procalcitonin (analyzer series Roche Cobas e801 module).

### Removal of LPS from murine serum (ex vivo test)

Four-week-old female CD1 mice (Envigo) weighing 20–25 g were used for the experiment. Only female mice were used for better group management. The experiment was designed with 2 animal groups of 6 mice. The experiment was performed in duplicate.

All animals had comparable weight; for these reasons, no criteria were applied to randomize the animals. Confounders were not controlled because they were sacrificed at the same time.

All precautions were used to minimize animal suffering. The animals were sedated before sacrifice.

No adverse events were reported during the experiment.

The animals never exhibited any conditions of discomfort that needed to be sacrificed. The animals were monitored hourly from treatment to sacrifice to note possible signs of animal discomfort.

LPS from *E. coli* (Serotype O111:K58(B4):H-) (Sigma-Aldrich) at 0.05 mg/kg was inoculated to simulate true sepsis in the mice.

Blood was drawn 4 hours after LPS injection, when the mice began to manifest the first signs of sepsis, showing slight slowing of movements and scruffy fur.

The samples were collected and processed the same day. Blood was drawn into tubes without an anticoagulant to obtain serum by centrifugation. Serum was filtered with a device containing 2 mL of agarose beads functionalized with 2 mg SET-M33 peptide. It was then recirculated for 2 h at a flow of 1 mL/min. After the passage of the serum in the circuit, endotoxin was quantified by the LAL test according to kit instructions. These experiments were executed adopting the ‘four Rs’ principles (Reduction, Refinement, Replacement and Responsibility). All procedures involving animals were approved by the Italian Ministry of Health, 14 January 2016, Protocol 34/2016-PR.

## Results

### Peptide production

**Fig. 1:**
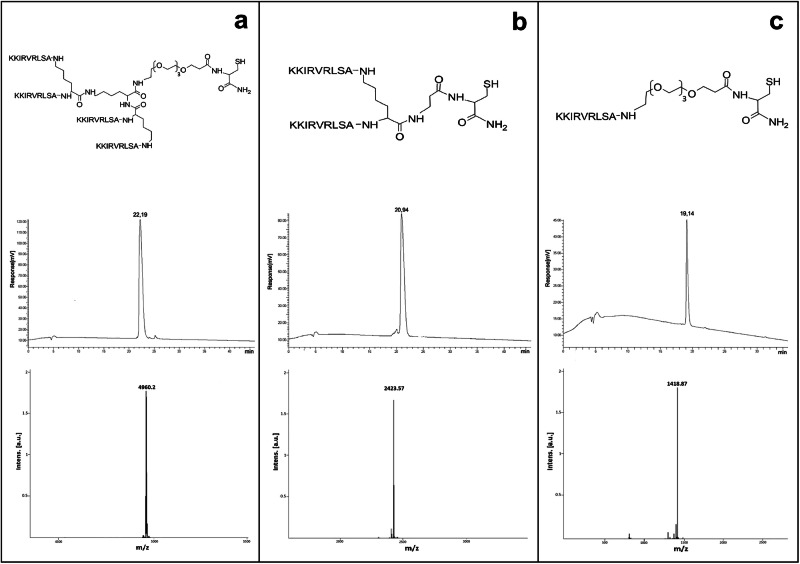
HPLC and MS profiles of SET-M33 peptide in three polymeric forms. **a** Tetrameric peptide, **b** dimeric peptide, and **c** monomeric peptide. TOP: peptide structure (the amino acids of the peptide sequences are indicated in one-letter code, not to scale); center: HPLC profile and retention time; bottom: MS profile and *m*/*z* ratio.

SET-M33 peptide was produced in tetra-branched (Fig. 1a), di-branched (Fig. 1b) and monomeric forms (Fig. 1c), all with a –SH group at the peptide C-terminal. The final purity of the three peptide forms was over 95%. The MS profiles showed a peak at molecular mass 4960.20 Da for the tetrameric peptide, 2423.57 Da for the dimeric peptide and 1418.87 Da for the monomeric peptide, in line with the expected molecular mass (Fig. 1a–c).

### Conjugation of SET-M33-cys with agarose beads

**Fig. 2:**
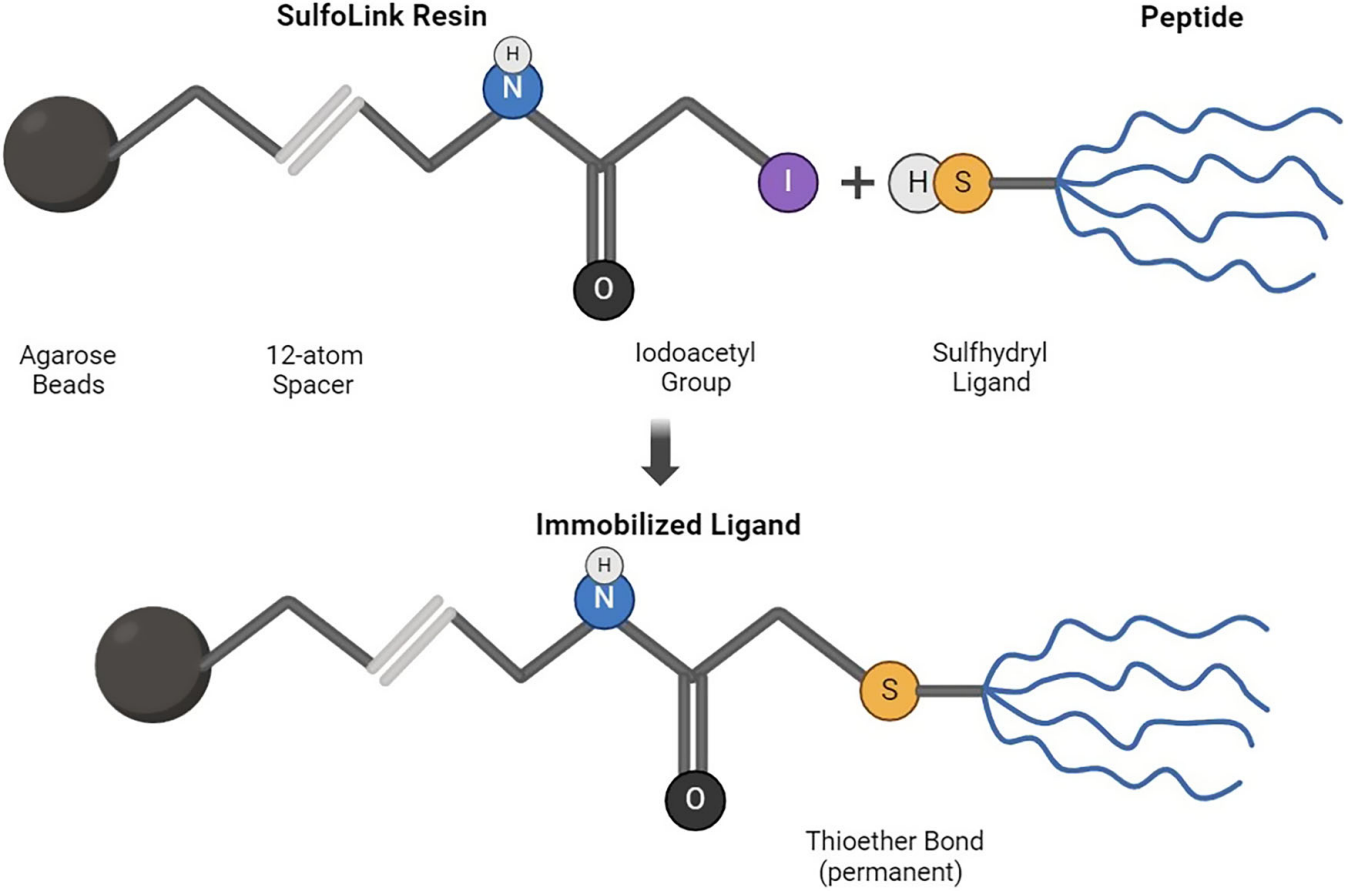
Mechanism of interaction and conjugation of the peptide with agarose beads. The tetrameric peptide is shown by way of example.

SulfoLink® coupling resin with agarose beads allowed conjugation of SET-M33-cys with a covalent bond. The iodoacetyl group reacted by nucleophilic substitution of iodine with a sulfur atom from a sulfhydryl group (–SH) in the side chain of cysteine at the peptide C-terminus, resulting in a stable thioether bond (Fig. 2).

### Comparison of bacterial removal between devices based on monomeric and polymeric peptides

In order to compare the bacterial removal capacity of the three forms of SET-M33, serum was spiked with a known amount of bacteria (*E. coli* ATCC 33780™), and then processed in devices containing monomeric, dimeric or tetrameric peptides. In order to compare the same number of interactive sites, the 2× concentrated dimer and 4× concentrated monomer were also tested. After incubation with the resin, serum was spread on LB agar plates and incubated overnight at 37 °C.

**Fig. 3:**
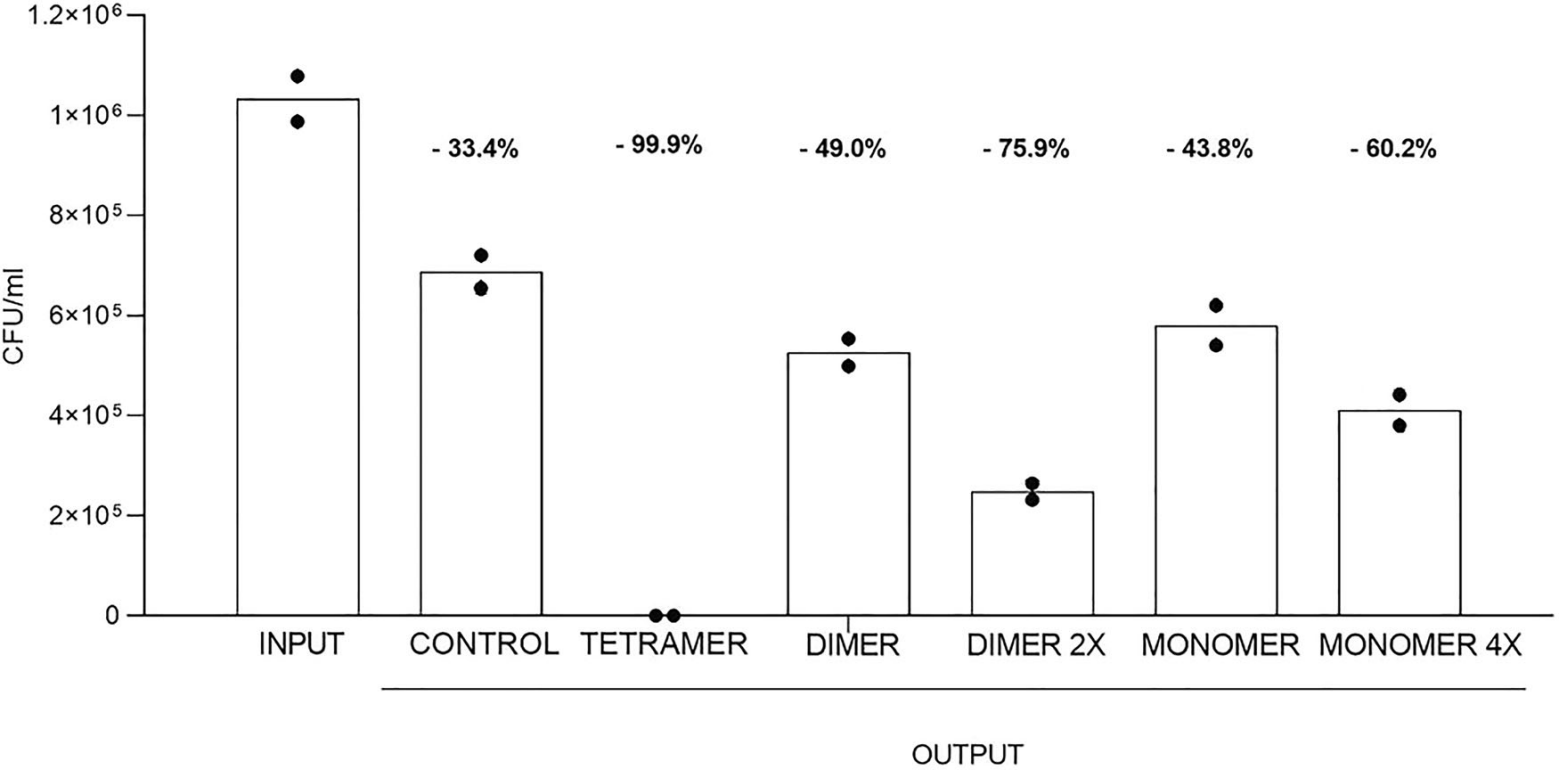
Histograms of bacterial removal with devices containing the peptide with different polymeric structures. The number of bacteria is indicated in the figure as colony forming unit (CFU). INPUT: number of bacteria in serum before incubation in the device (*n* = 2); CONTROL OUTPUT: number of bacteria in serum after incubation in device without peptide (*n* = 2); TETRAMER OUTPUT: number of bacteria in serum after incubation in device containing SET-M33 in tetrameric form (*n* = 2); DIMER OUTPUT and 2X DIMER OUTPUT: number of bacteria in serum after incubation in device containing SET-M33 in dimeric form, at the same concentration as tetramer or 2X, respectively (*n* = 2); MONOMER OUTPUT and 4X MONOMER OUTPUT: number of bacteria in serum after incubation in device containing SET-M33 in monomeric form, at the same concentration as tetramer or 4X, respectively (*n* = 2). Columns represent group means.

Peptide in tetrameric form showed a significantly stronger removal rate than peptides in dimeric and monomeric forms (Fig. 3). 99.9% bacterial removal was observed for devices with tetrameric peptide, 49.0% with dimer at the same concentration as tetramer, 75.9% with 2× dimer, 43.8% with monomer at the same concentration as tetramer, and 60.2% for devices with 4× monomer. Partial removal of bacteria (33.4%) was observed in the device without peptide (CONTROL) because some bacteria remained entangled in the resin during incubation.

Since the tetrameric peptide resulted the best decoy in terms of removal activity, the device with this peptide was used for further characterizations.

### Stability test

The efficacy of the device was evaluated by testing its ability to remove bacteria (*E. coli* ATCC 33780™) from serum immediately after immobilization of the peptide in the device (time zero) and after storage of the device for 30 days at 4 °C and at room temperature.

**Fig. 4:**
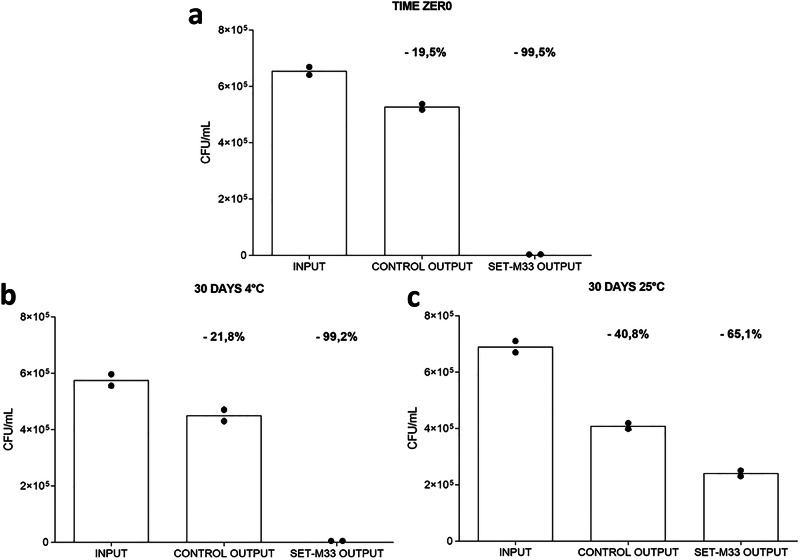
Stability of devices under different temperature conditions. The number of bacteria is indicated in the figure as colony forming unit (CFU). **a** Histograms of bacterial removal by devices at time zero (*n* = 2) and **b** by devices stored for 30 days at 4 °C (*n* = 2*)* and **c** at room temperature (*n* = 2). The INPUT histogram indicates serum containing bacteria before incubation, CTR OUTPUT represents serum in the device without peptide and SET-M33 OUTPUT represents serum after incubation in the device with the peptide. Columns represent a group.

The device tested at time zero showed a 99.5% reduction in bacteria (Fig. 4a). In the second test, 30 days after conjugation of the peptide with agarose beads, the device stored at 4 °C showed a 99.2% reduction in bacteria (Fig. 4b), while the device stored at room temperature showed a 65.1% reduction (Fig. 4c). Partial removal of bacteria (19.5% at time zero and 21.8% and 40.8% at 30 days) was observed in the control device (CTR OUTPUT) because some bacteria remained entangled in the resin during incubation.

### Removal of LPS and LTA from human serum

**Fig. 5:**
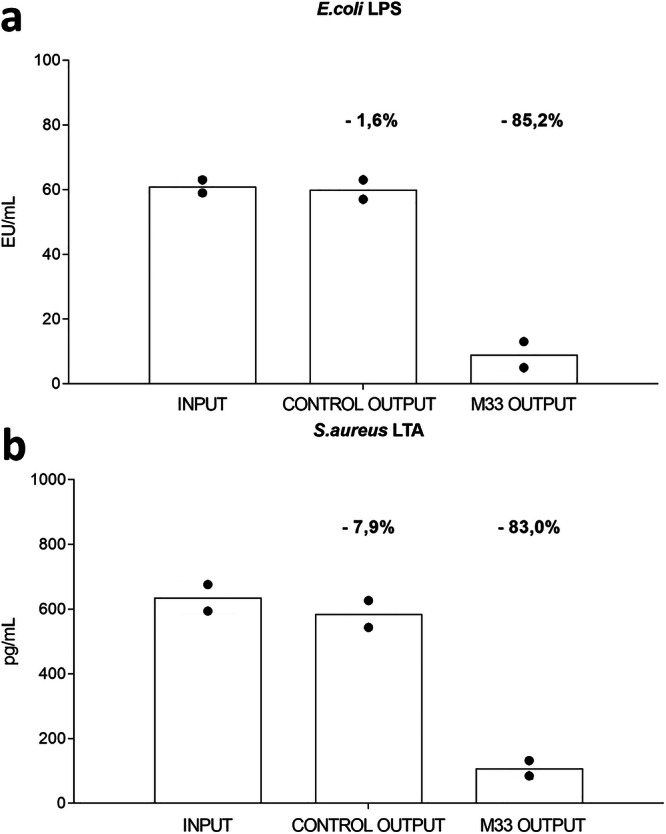
Removal of LPS and LTA from human serum. **a** Histograms of *E. coli* lipopolysaccharide (LPS) removal from human serum diluted 1:10 in physiological solution (*n* = 2). **b** Histograms of *S. aureus* (lipoteichoic acid) LTA removal from human serum diluted 1:10 in physiological solution (*n* = 2). Lipopolysaccharide is shown in Endotoxin Units (EU). LPS (75 EU/mL) and LTA (500 pg/mL) were used in these experiments. Columns represent a group.

The prototype device with tetrameric SET-M33 was also used to evaluate the removal of LPS (*E. coli* Serotype O111:K58[B4], Sigma, St. Louis, MO, 75 EU/mL) and LTA (*S. aureus*, MyBiosource, 500 pg/mL) from human serum. A cartridge loaded with non-conjugated resin was used as a negative control. Human serum from healthy individuals spiked with LPS or LTA was incubated under constant rocking for 2 h with the resin. Agarose-SET-M33 resin removed 85.2% of LPS (1.6% control) (Fig. 5a) and 83% of LTA (7.9% control) (Fig. 5b).

### Ex vivo removal of LPS

In order to evaluate the removal capacity in real sepsis, we set up an ex vivo experiment where serum of mice challenged with LPS was processed in the SET-M33 device.

**Fig. 6:**
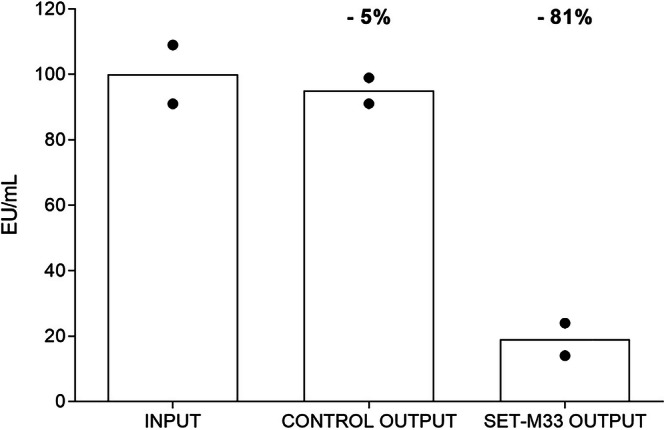
Reduction of LPS content in the serum of mice previously challenged with 0.05 mg/kg LPS. Lipopolysaccharide is shown in Endotoxin Units (EU). An 81% difference between unfiltered serum (INPUT) and serum collected after filtration (SET-M33 OUTPUT) is shown, corresponding to EU/mL values of 48.3 and 8.9, respectively (*n*  =  6 mice per group). Columns represent group means.

Mice were inoculated with 0.05 mg/kg LPS (*E. coli* Serotype O111:K58 B4) and after 24 h, sacrificed for blood withdrawal. The serum obtained was incubated in the prototype SET-M33 device for 2 h under rotation at room temperature. A LAL test was performed on the serum before and after filtration. A reduction of 81% in LPS was recorded (Fig. 6). corresponding to EU/mL values of 48.3 (input) and 8.9 (output).

### Removal of live bacteria from human serum

The prototype SET-M33 device was used to remove live bacteria from human serum. The bacteria used in the experiments were *E. coli ATCC 33780*, representing Gram-negative and *S. aureus* USA300, representing Gram-positive bacteria. Serum samples spiked with known amounts of bacteria were processed in the device, then appropriately diluted and plated in suitable media for colony growth.

**Fig. 7:**
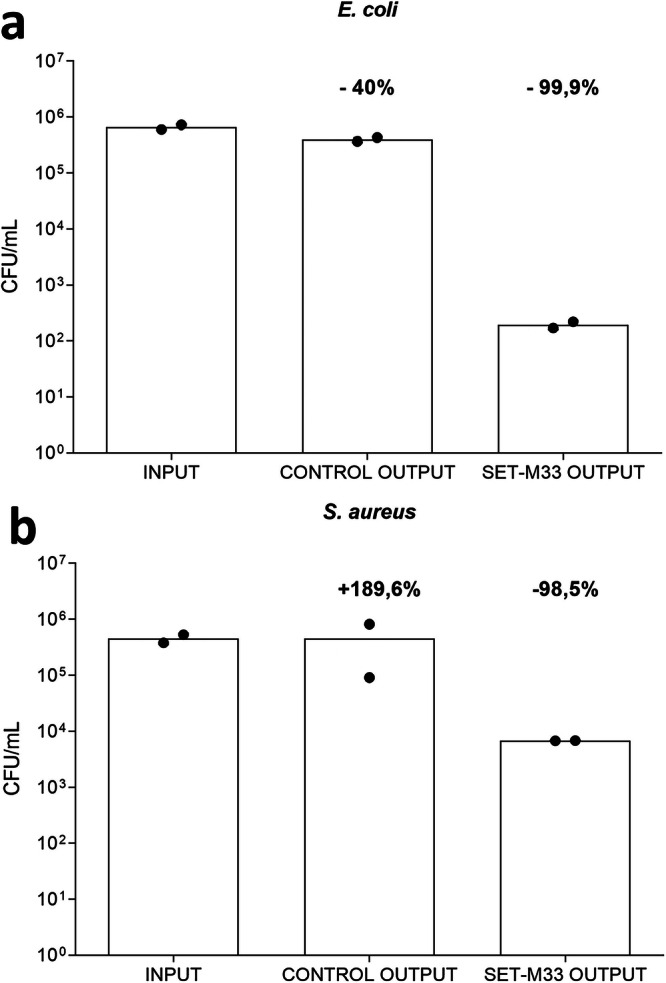
Removal of *E. coli* ATCC 33780 (a) and *S. aureus* USA 300 (b) from human serum. The number of bacteria is indicated in the figure as Colony Forming Unit (CFU). **a** Histograms of *E. coli* live bacteria removal from human serum (*n* = 2). **b** Histograms of *S. aureus* removal from human serum (*n* = 2). SET-M33 OUTPUT graphs refer to devices containing SET-M33, while CONTROL OUTPUT graphs refer to the same devices without SET-M33. The INPUT histograms refer to sera containing bacteria before passage through the device. The increase in *S. aureus* in the control column is indicated as a percentage, but is not evident on the logarithmic scale. Columns represent group means.

The results showed a 99.9% reduction in Gram-negative and a 98.5% reduction in Gram-positive bacteria in the SET-M33 device for the two bacterial species (Fig. 7). The control device (cartridge with agarose resin without the peptide) showed different behavior for the two bacteria. *E. coli* remained partially trapped in the agarose matrix, producing non-specific removal of 40% of the bacteria (Fig. 7a). The colony count of *S. aureus* increased by 89.6% (Fig. 7b). *S. aureus* presumably found a favorable environment for growth in this matrix in the absence of the peptide.

### Capillary electrophoresis and blood chemistry parameters

Human serum from pools of healthy subjects and sepsis patients, before and after incubation with devices containing or not containing SET-M33, was analyzed by capillary electrophoresis. The blood pool used in the experiments is completely anonymous. No informed consent or request for Institutional Review Board (IRB) approval was required because all samples were intended for disposal. The protein content of healthy serum after passage in the device was practically unmodified (Table 1). The same was found for septic serum, except for the beta-2 globulin fraction, which showed a sharp reduction (about 40%). The beta-2 fraction contains important inflammatory components, such as complement C3 and C4 proteins, C-reactive protein (CPR) and clotting factors, involved in the progression of sepsis^46^.

**Table 1:** Electrophoresis of serum proteins

	Blood serum proteins electrophoresis	Reference values	Input	Ctr device (output)	SET-M33 device (output)
Serum of healthy subjects	Albumin	53–66%	61.7	60.2	63.5
	Alpha-1	1.9–4.5%	4	3.8	3.6
	Alpha-2	6.5–13%	9.8	10.6	9.3
	Beta-1	4–6%	6.3	6.3	5.9
	Beta-2	1–3%	4.4	3.8	3
	Gamma	11.1-18.5%	13.8	15.3	14.7
Serum of patients with sepsis	Albumin	53–66%	51.7	52.6	54.8
	Alpha-1	1.9–4.5%	8.2	7.4	7.9
	Alpha-2	6.5–13%	13.4	13.9	11.5
	Beta-1	4–6%	4.9	4.9	5.7
	Beta-2	1–3%	6.2	5.3	3.7
	Gamma	11.1–18.5%	15.6	15.9	16.4

Percentages of blood proteins in pools of serum from healthy subjects and patients with sepsis before and after passage through control (CTR) and SET-M33 devices.

### Removal of C-reactive protein

**Fig. 8:**
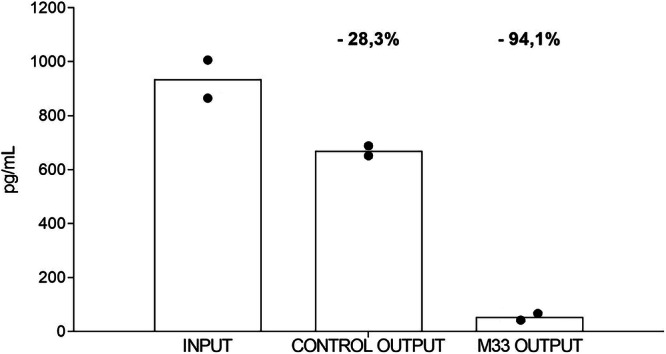
Reduction of CRP. The INPUT column indicates the amount of C-reactive protein (CRP) in serum before passage through the cartridges. The CONTROL column indicates the amount of CRP after passage in the cartridge without peptide (*n* = 2). The SET-M33 column indicates the amount of CRP after passage in the SET-M33 cartridge (*n* = 2). The removal percentages are indicated above the CTR and SET-M33 columns. Columns represent group means.

C-reactive protein (CRP) proved to be the protein that decreased most after passage of septic serum in the SET-M33 cartridge (Table 2). This removal was specific by virtue of the direct binding of the protein to the SET-M33 peptide in the resin. Figure 8 shows that CRP decreased by 94.1% in the cartridge containing the peptide, whereas only poor non-specific removal occurred with the control cartridge (28.3%). Procalcitonin (PCT) was also removed, albeit mainly due to non-specific trapping in the resin (not shown). However, this non-specific abatement of PCT was very evident when the protein reached high concentrations, as in septic serum, whereas it was not at all evident in healthy serum, where the PCT levels are low by default (Table 2).

**Table 2:** Electrophoretic comparison of blood serum proteins

	Clinical data	Reference values	Input	Ctr device (output)	SET-M33 devices (output)
Serum of healthy subjects	C-reactive protein (mg/dL)	<0.5	0.09	0.06	<0.03
	Procalcitonin (ng/mL)	<0.05	0.02	0.02	0.02
	Total Protein (g/dL)	6.4–8.3	7	6.9	5.8
	Albumin (g/dL)	3.5–5.5	4.7	4.6	3.9
Serum of patients with sepsis	C-reactive protein (mg/dL)	<0.5	10.18	8.53	4.41
	Procalcitonin (ng/mL)	<0.05	8.21	5.67	4.47
	Total Protein (g/dL)	6.4–8.3	5.2	4.3	3.4
	Albumin (g/dL)	3.5–5.5	3	2.4	1.9

Comparison of blood serum protein in pools of serum from healthy subjects and patients with sepsis, before and after passage through control (CTR) and SET-M33 devices.

## Discussion

Sepsis is a systemic syndrome that can result in extremely low blood pressure, organ failure and death. Sepsis has always been a research topic because of its complexity and consequences for patients and healthcare systems. The extensive use of antibiotics has led to a great reduction in cases of sepsis and deaths, but the proliferation of multidrug-resistant bacteria in recent years has created a new state of emergency for our species. To treat sepsis, besides research for new antimicrobial drugs, there has been active R&D of medical devices for the removal of triggers of sepsis. These devices are “filters” with adsorptive properties used in extracorporeal circulation systems. Among the many applications of these devices, a major goal is the effective removal of bacteria and endotoxins. Commercially available medical devices capable of removing live bacteria (Seraph 100^47^), endotoxins (Toraymyxin^48^; Matisse^49^; Alteco^50^) and cytokines (Cytosorb^51^; oXiris^52^) are in clinical use, but none have yet proved able to remove different pathological components, such as LPS, LTA and live bacteria, simultaneously. This is one reason why clinical outcomes from the use of these devices are not uniform and too often not resolutive in sepsis patients^20,35^. Many studies involving some of these devices have been terminated prematurely due to a lack of improvement in 28-day mortality, vasopressor requirements, ventilation days and ICU length of stay compared to conventional treatments^36,37^.

SET-M33 is an antimicrobial peptide extensively described as a possible new antibacterial agent^39,53,54^. It has excellent antimicrobial characteristics, anti-inflammatory activity by selective neutralization of LPS, low hemolytic activity, lack of immunogenicity, and an ability to eradicate biofilm^39–42,53–56^. Its strong positive net charge allows it to selectively interact with anionic bacterial membranes and other negatively charged structures, such as LPS in Gram-negative or LTA in Gram-positive bacteria.

For all these reasons, we selected SET-M33 as a promising candidate for the construction of a medical device for the simultaneous selective removal of bacterial triggers in the treatment of sepsis. Here, we described the engineering of the peptide SET-M33 for immobilization on agarose beads in a prototype device designed for use in extracorporeal circulation (plasmapheresis). SET-M33 was studied in different polymeric structures. The tetrameric form proved to be the best polymer in terms of removal efficiency and was chosen for further characterization.

In vitro and ex vivo experiments demonstrated that this medical device has a similar removal capacity for Gram-negative and Gram-positive bacteria. The antibacterial action of SET-M33 is based on interaction with the bacterial surface and membrane disruption. This may cause release of bacterial components, including the PAMPs that most promote progression of sepsis, as well as LPS, LTA and Lipoproteins associated with LTA^57–61^. The prototype of this device proved able to remove and neutralize LPS and LTA, and evidently also Lipoproteins associated with LTA, from serum. At the same time, it strongly abated inflammatory factors like CRP and PCT, two important indicators of the progression of sepsis. However, a sharp rise in CRP levels is not only a diagnostic parameter in sepsis patients, but is involved in killing bacteria through opsonization with complement activation and cell lysis. Unfortunately, where inflammatory processes are out of control, as in sepsis, strong overexpression of CRP can also cause significant damage to host tissues and organs, contributing to the progression of sepsis and septic shock^46^. The specific reduction of CRP, along with simultaneous removal of bacterial cells and toxins, therefore becomes a major point of strength of this medical device, because it directly reduces all the processes of inflammatory activation and progression that occur in sepsis when antibiotics and supportive therapies are failing. The peptide SET-M33 appears to be a perfect decoy for eliminating triggers of sepsis from the blood of patients in ICUs, even in situations where superinfections of Gram-negative and Gram-positive bacteria are involved. The prototype here described is, as far as we are aware, the first device to simultaneously offer all these activities in the same circulation event.

### Reporting summary

Further information on research design is available in the Nature Portfolio Reporting Summary linked to this article.

## Supplementary information

Reporting summary

## Data Availability

The source data for Figs. 3–8 are available in the Figshare repository (https://figshare.com/s/5b5a9e9dc0d018da3f2a), 10.6084/m9.figshare.27160296. The Figshare repository contains the data (means, standard deviations and statistical methods) from which the graphs were obtained. The raw datasets generated during the current study are available from the corresponding author on reasonable request.

## References

[1] Singer, M. et al. The third international consensus definitions for sepsis and septic shock (Sepsis-3). *JAMA* **315**, 801–810 (2016). 26903338 10.1001/jama.2016.0287 PMC4968574

[2] Srzić, I., Nesek Adam, V. & Tunjić Pejak, D. Sepsis definition: what’s new in the treatment guidelines. *Acta Clin. Croatia* **61**, 67–72 (2022). 10.20471/acc.2022.61.s1.11 PMC9536156 36304809

[3] Rudd, K. E. et al. Global, regional, and national sepsis incidence and mortality, 1990–2017: analysis for the Global Burden of Disease Study.*Lancet* **395**, 200–211 (2020). 31954465 10.1016/S0140-6736(19)32989-7 PMC6970225

[4] Davis, J. S., He, V., Anstey, N. M. & Condon, J. R. Long term outcomes following hospital admission for sepsis using relative survival analysis: a prospective cohort study of 1,092 patients with 5 years follow up. *PLoS ONE* **9**, e112224 (2014). 25486241 10.1371/journal.pone.0112224 PMC4259299

[5] Rubin R., Strayer D. S., Rubin E. Rubin's Pathology: *Clinicopathologic Foundations of Medicine* VII edn (Wolters Kluwer-Lippincott Williams & Wilkins, 2014).

[6] Abbas A. K., Lichtman A. H., Pillai S. *Cellular and Molecular Immunology* X edn (Elsevier, 2021).

[7] Wang, J., Wang, H., Liu, W., Zhang, D. & Guo, S. Assessment values of procalcitonin, lactic acid, and disease severity scores in patients with sepsis. *Zhonghua Wei Zhong Bing Ji Jiu Yi Xue* **31**, 938–941 (2019). 31537215 10.3760/cma.j.issn.2095-4352.2019.08.005

[8] Hao, C., Hu, Q., Zhu, L., Xu, H. & Zhang, Y. Combined prognostic value of serum lactic acid, procalcitonin and severity score for short-term prognosis of septic shock patients. *Zhonghua Wei Zhong Bing Ji Jiu Yi Xue* **33**, 281–285 (2021). 33834968 10.3760/cma.j.cn121430-20201113-00715

[9] Zhao, M. & Duan, M. Lactic acid, lactate clearance and procalcitonin in assessing the severity and predicting prognosis in sepsis. *Zhonghua Wei Zhong Bing Ji Jiu Yi Xue* **32**, 449–453 (2020). 32527351 10.3760/cma.j.cn121430-20200129-00086

[10] Durrance, R. J. et al. Marked elevation in serum procalcitonin levels do not correlate with severity of disease or mortality in hospitalized patients: a retrospective study. *Biomark. Insights* **15**, 1177271920917941 (2020). 32476970 10.1177/1177271920917941 PMC7232048

[11] Yu, H. et al. Combining procalcitonin with the qSOFA and sepsis mortality prediction. *Medicine* **98**, e15981 (2019). 10.1097/MD.0000000000015981 PMC6571275 31169735

[12] Qiu, X., Lei, Y. & Zhou, R. SIRS, SOFA, qSOFA, and NEWS in the diagnosis of sepsis and prediction of adverse outcomes: a systematic review and meta-analysis. *Expert Rev. Anti-infect. Ther.* **21**, 891–900 (2023). 37450490 10.1080/14787210.2023.2237192

[13] Lin, E. & Lowry, S. F. The human response to endotoxin. *Sepsis* **2**, 255–262 (1998).

[14] Beutler, B. Endotoxin, toll-like receptor 4, and the afferent limb of innate immunity. *Curr. Opin. Microbiol.* **3**, 23–28 (2000). 10679425 10.1016/s1369-5274(99)00046-6

[15] Castellheim, A., Brekke, O.-L., Espevik, T., Harboe, M. & Mollnes, T. E. Innate immune responses to danger signals in systemic inflammatory response syndrome and sepsis. *Scand. J. Immunol.* **69**, 479–491 (2009). 19439008 10.1111/j.1365-3083.2009.02255.x

[16] Cohen, J. The immunopathogenesis of sepsis. *Nature.* **420**, 885–891 (2002). 12490963 10.1038/nature01326

[17] Kang, S. S., Sim, J. R., Yun, C. H. & Han, S. H. Lipoteichoic acids as a major virulence factor causing inflammatory responses via Toll-like receptor 2. *Arch. Pharm. Res.* **39**, 1519–1529 (2016). 27498542 10.1007/s12272-016-0804-y

[18] Al-Kader, D. A. et al. Systematic review on the effects of prompt antibiotic treatment on survival in septic shock and sepsis patients in different hospital settings. *Cureus.* **14**, e32405 (2022). 36636534 10.7759/cureus.32405 PMC9831358

[19] Shen, X. F., Cao, K., Jiang, J. P., Guan, W. X. & Du, J. F. Neutrophil dysregulation during sepsis: an overview and update. *J. Cell. Mol. Med.* **21**, 1687–1697 (2017). 28244690 10.1111/jcmm.13112 PMC5571534

[20] Jarczak, D., Kluge, S. & Nierhaus, A. Sepsis-pathophysiology and therapeutic concepts. *Front. Med.* **8**, 628302 (2021). 10.3389/fmed.2021.628302 PMC8160230 34055825

[21] Martin, G. S., Mannino, D. M., Eaton, S. & Moss, M. The epidemiology of sepsis in the United States from 1979 through 2000. *New Engl. J. Med.* **348**, 1546–1554 (2023). 10.1056/NEJMoa022139 12700374

[22] Sriskandan, S. & Cohen, J. Gram-positive sepsis. Mechanisms and differences from Gram-negative sepsis. *Infect. Dis. Clin. N. Am.* **13**, 397–412 (1999). 10.1016/s0891-5520(05)70082-9 10340174

[23] Danner, R. L. et al. Endotoxemia in human septic shock. *Chest.* **99**, 169–175 (1991). 1984950 10.1378/chest.99.1.169

[24] Venet, C. et al. Endotoxaemia in patients with severe sepsis or septic shock. *Intensive Care Med.* **26**, 538–544 (2000). 10923727 10.1007/s001340051201

[25] Hurley, J. C. Endotoxemia and Gram-negative bacteremia as predictors of outcome in sepsis: a meta-analysis using ROC curves. *J. Endotoxin Res.* **9**, 271–279 (2003). 14577843 10.1179/096805103225002511

[26] Esteban, E., Ferrer, R., Alsina, L. & Artigas, A. Immunomodulation in sepsis: the role of endotoxin removal by Polymyxin B-immobilized cartridge. *Mediat. Inflamm*. **2013**, 507539 (2013). 10.1155/2013/507539 PMC3819752 24249974

[27] Malard, B., Lambert, C. & Kellum, J. A. In vitro comparison of the adsorption of inflammatory mediators by blood purification devices. *Intensive Care Med. Exp.* **6**, 12 (2018). 29728790 10.1186/s40635-018-0177-2 PMC5935601

[28] Cecchi, M., Ulsamer, A. & Villa, G. Oxiris membrane in sepsis and multiple organ failure. *Contrib. Nephrol.* **200**, 55–65 (2023). 37263191 10.1159/000527355

[29] Tani, T., Shimizu, T., Tani, M., Shoji, H. & Endo, Y. Anti-endotoxin properties of Polymyxin B-immobilized fibers. *Adv. Exp. Med. Biol.* **1145**, 321–341 (2019). 31364085 10.1007/978-3-030-16373-0_19 PMC7123644

[30] Borthwick, E. M. et al. High-volume haemofiltration for sepsis in adults. *Cochrane Database Syst. Rev.* **1**, CD008075 (2017). 28141912 10.1002/14651858.CD008075.pub3 PMC6464723

[31] Paul, R. et al. Multicentered prospective investigator-initiated study to evaluate the clinical outcomes with extracorporeal cytokine adsorption device (CytoSorb®) in patients with sepsis and septic shock. *World J. Crit. Care Med.* **10**, 22–34 (2021). 33505870 10.5492/wjccm.v10.i1.22 PMC7805252

[32] Saldaña-Gastulo, J. J. C., Llamas-Barbarán, M., Coronel-Chucos, L. G. & Hurtado-Roca, Y. Cytokine hemoadsorption with CytoSorb® in patients with sepsis: a systematic review and meta-analysis. *Crit. Care Sci.* **35**, 217–225 (2023). 37712812 10.5935/2965-2774.20230289-en PMC10406402

[33] Adamik, B., Zielinski, S., Smiechowicz, J. & Kübler, A. Endotoxin elimination in patients with septic shock: an observation study. *Arch. Immunol. Ther. Exp.* **63**, 475–483 (2015). 10.1007/s00005-015-0348-8 PMC4633444 26093653

[34] Sazonov, V. et al. Case Series: Efficacy and safety of hemoadsorption with HA 330 adsorber in septic pediatric patients with cancer. *Front. Pediatr.* **9**, 672260 (2021). 34178889 10.3389/fped.2021.672260 PMC8225958

[35] Feng, Y., Peng, J. & Peng, Z. Blood purification in sepsis and systemic inflammation. *Curr. Opin.Clin. Care* **27**, 582–586 (2021). 10.1097/MCC.0000000000000890 34581298

[36] Honoré, P. M. et al. New insights regarding rationale, therapeutic target and dose of hemofiltration and hybrid therapies in septic acute kidney injury. *Blood Purif.* **33**, 44–51 (2012). 22179226 10.1159/000333837

[37] Livigni, S. et al. Efficacy of coupled plasma filtration adsorption (CPFA) in patients with septic shock: a multicenter randomised controlled clinical trial. *BMJ Open* **4**, e003536 (2014). 24401721 10.1136/bmjopen-2013-003536 PMC3902195

[38] Pini, A. et al. A novel tetrabranched antimicrobial peptide that neutralizes bacterial lipopolysaccharide and prevents septic shock in vivo. *FASEB J.* **24**, 1015–1022 (2010). 19917670 10.1096/fj.09-145474

[39] Brunetti, J. et al. In vitro and in vivo efficacy, toxicity, bio-distribution and resistance selection of a novel antibacterial drug candidate. *Sci. Rep.* **6**, 26077 (2016a). 27169671 10.1038/srep26077 PMC4864329

[40] Cresti, L. et al. In Vivo efficacy and toxicity of an antimicrobial peptide in a model of endotoxin-induced pulmonary inflammation. *Int. J. Mol. Sci.* **24**, 7967 (2023). 37175674 10.3390/ijms24097967 PMC10178222

[41] Cresti, L. et al. Safety evaluations of a synthetic antimicrobial peptide administered intravenously in rats and dogs. *Sci. Rep.* **12**, 19294 (2022a). 36369523 10.1038/s41598-022-23841-2 PMC9652379

[42] Van der Weide, H. et al. Investigations into the killing activity of an antimicrobial peptide active against extensively antibiotic-resistant K. pneumoniae and *P. aeruginosa*. *Biochim. Biophys. Acta* **1859**, 1796–1804 (2017). 10.1016/j.bbamem.2017.06.001 28583831

[43] Quercini, L. et al. An antimicrobial molecule mitigates signs of sepsis in vivo and eradicates infections from lung tissue. *FASEB J.* **34**, 192–207 (2020). 31914681 10.1096/fj.201901896RR

[44] Dyzenhaus, S. et al. MRSA lineage USA300 isolated from bloodstream infections exhibit altered virulence regulation. *Cell Host Microbe* **31**, 228–242.e8 (2023). 36681080 10.1016/j.chom.2022.12.003 PMC9911362

[45] Falciani, C. et al. Isomerization of an antimicrobial peptide broadens antimicrobial spectrum to Gram-positive bacterial pathogens. *PLoS ONE* **7**, e46259 (2012). 23056272 10.1371/journal.pone.0046259 PMC3462775

[46] Sproston, N. R. & Ashworth, J. J. Role of C-reactive protein at sites of inflammation and infection. *Front. Immunol.* **9**, 754 (2018). 29706967 10.3389/fimmu.2018.00754 PMC5908901

[47] Eden, G. et al. Safety and efficacy of the Seraph® 100 Microbind® Affinity Blood Filter to remove bacteria from the blood stream: results of the first in human study. *Crit. Care* **26**, 181 (2022). 35715801 10.1186/s13054-022-04044-7 PMC9205040

[48] Shoji, H. Extracorporeal endotoxin removal for the treatment of sepsis: endotoxin adsorption cartridge (Toraymyxin). *Ther. Apher. Dial.* **7**, 108–114 (2003). 12921125 10.1046/j.1526-0968.2003.00005.x

[49] Staubach, K. H., Boehme, M., Zimmermann, M. & Otto, V. A new endotoxin adsorption device in Gram-negative sepsis: use of immobilized albumin with the MATISSE adsorber. *Transfus. Apher. Sci.* **29**, 93–98 (2003). 12877899 10.1016/s1473-0502(03)00100-9

[50] Lipcsey, M. et al. Endotoxin removal in septic shock with the Alteco LPS Adsorber was safe but showed no benefit compared to placebo in the double-blind randomized controlled trial—the asset study. *Shock* **54**, 224–231 (2020). 31880758 10.1097/SHK.0000000000001503

[51] Hinz, B. et al. CytoSorb, a novel therapeutic approach for patients with septic shock: a case report. *Int. J. Artif. Organs* **38**, 461–464 (2015). 26349530 10.5301/ijao.5000429

[52] Broman, M. E., Hansson, F., Vincent, J. L. & Bodelsson, M. Endotoxin and cytokine reducing properties of the oXiris membrane in patients with septic shock: a randomized crossover double-blind study. *PLoS ONE* **14**, e0220444 (2019). 31369593 10.1371/journal.pone.0220444 PMC6675097

[53] Cresti, L. et al. Inhalable polymeric nanoparticles for pulmonary delivery of antimicrobial peptide SET-M33: antibacterial activity and toxicity in vitro and in vivo. *Pharmaceutics* **15**, 3 (2022b). 36678633 10.3390/pharmaceutics15010003 PMC9863998

[54] Pini, A., Falciani, C. & Bracci, L. Branched peptides as therapeutics. *Curr. Protein Peptide Sci.* **9**, 468–477 (2008). 18855698 10.2174/138920308785915227

[55] Brunetti, J. et al. Immunomodulatory and anti-inflammatory activity in vitro and in vivo of a novel antimicrobial candidate. *J. Biol. Chem.* **291**, 25742–25748 (2016b). 27758868 10.1074/jbc.M116.750257 PMC5207269

[56] Cresti, L., Cappello, G. & Pini, A. Antimicrobial peptides towards clinical application—a long history to be concluded. *Int. J. Mol. Sci.* **25**, 4870 (2024). 29. 38732089 10.3390/ijms25094870 PMC11084544

[57] Zähringer, U., Lindnera, B., Inamuraa, S., Heineb, H. & Alexandera, C. TLR2—promiscuous or specific? A critical re-evaluation of a receptor expressing apparent broad specificity. *Immunobiology.* **213**, 205–224 (2008). 18406368 10.1016/j.imbio.2008.02.005

[58] Martinez De Tejada, G. et al. Lipoproteins/peptides are sepsis inducing toxins from bacteria that can be neutralized by synthetic anti-endotoxin peptides. *Sci. Rep.* **5**, 14292 (2015). 10.1038/srep14292 PMC4585737 26390973

[59] Rockel, C. & Hartung, T. Systematic review of membrane components of Gram positive bacteria responsible as pyrogens for inducing human monocyte/macrophage cytokine release. *Front. Pharmacol.* **3**, 56 (2012). 22529809 10.3389/fphar.2012.00056 PMC3328207

[60] Hashimoto, M. et al. Not lipoteichoic acid but lipoproteins appear to be the dominant immunobiologically active compounds in *Staphylococcus aureus*. *J. Immunol.* **177**, 3162–3169 (2006). 16920954 10.4049/jimmunol.177.5.3162

[61] Gisch, N. et al. Structural reevaluation of *Streptococcus pneumonia* lipoteichoic acid and new Insights into its immunostimulatory potency. *J. Biol. Chem.* **288**, 15654–15667 (2013). 23603911 10.1074/jbc.M112.446963 PMC3668725

