# Supplementary material for: A peptide-based medical device for the selective removal of inflammatory factors from the blood of people with sepsis
Source: Commun Med (Lond). 2025 Oct 27;5:436. doi: 10.1038/s43856-025-01187-w (PMC12559756; doi:10.1038/s43856-025-01187-w)
Supplement: Supplementary file 1 — Reporting summary [file 43856_2025_1187_MOESM1_ESM.pdf]

nature portfolio

Corresponding author(s): Alessandro Pini
COMMSMED-24-1038C

Last updated by author(s): Mar 31, 2025

# Reporting Summary

Nature Portfolio wishes to improve the reproducibility of the work that we publish. This form provides structure for consistency and transparency in reporting. For further information on Nature Portfolio policies, see our Editorial Policies and the Editorial Policy Checklist.

## Statistics

For all statistical analyses, confirm that the following items are present in the figure legend, table legend, main text, or Methods section.

| n/a | Confirmed | |
|---|---|---|
| ☒ | ☐ | The exact sample size (*n*) for each experimental group/condition, given as a discrete number and unit of measurement |
| ☐ | ☒ | A statement on whether measurements were taken from distinct samples or whether the same sample was measured repeatedly |
| ☒ | ☐ | The statistical test(s) used AND whether they are one- or two-sided<br>*Only common tests should be described solely by name; describe more complex techniques in the Methods section.* |
| ☒ | ☐ | A description of all covariates tested |
| ☐ | ☒ | A description of any assumptions or corrections, such as tests of normality and adjustment for multiple comparisons |
| ☐ | ☒ | A full description of the statistical parameters including central tendency (e.g. means) or other basic estimates (e.g. regression coefficient) AND variation (e.g. standard deviation) or associated estimates of uncertainty (e.g. confidence intervals) |
| ☐ | ☒ | For null hypothesis testing, the test statistic (e.g. $F$, $t$, $r$) with confidence intervals, effect sizes, degrees of freedom and $P$ value noted<br>*Give P values as exact values whenever suitable.* |
| ☒ | ☐ | For Bayesian analysis, information on the choice of priors and Markov chain Monte Carlo settings |
| ☒ | ☐ | For hierarchical and complex designs, identification of the appropriate level for tests and full reporting of outcomes |
| ☒ | ☐ | Estimates of effect sizes (e.g. Cohen's $d$, Pearson's $r$), indicating how they were calculated |

*Our web collection on statistics for biologists contains articles on many of the points above.*

## Software and code

Policy information about availability of computer code

| | |
|---|---|
| Data collection | GraphPad Prism for Windows version 8.4.2, GraphPad Software, San Diego, CA, USA. |
| Data analysis | Comparisons were made between the input group and the other individual groups. Significant differences between groups are indicated as follows: *** p < 0.001, ** p < 0.01 and * p < 0.05 |

For manuscripts utilizing custom algorithms or software that are central to the research but not yet described in published literature, software must be made available to editors and reviewers. We strongly encourage code deposition in a community repository (e.g. GitHub). See the Nature Portfolio guidelines for submitting code & software for further information.

## Data

Policy information about availability of data

All manuscripts must include a data availability statement. This statement should provide the following information, where applicable:

- Accession codes, unique identifiers, or web links for publicly available datasets
- A description of any restrictions on data availability
- For clinical datasets or third party data, please ensure that the statement adheres to our policy

Sera from healthy and septic patients was collected. The collection of sera was done anonymously by volunteer patients.

# Human research participants

Policy information about studies involving human research participants and Sex and Gender in Research.

| | |
|---|---|
| Reporting on sex and gender | NOT APPLICABLE |
| Population characteristics | NOT APPLICABLE |
| Recruitment | NOT APPLICABLE |
| Ethics oversight | NOT APPLICABLE |

Note that full information on the approval of the study protocol must also be provided in the manuscript.

# Field-specific reporting

Please select the one below that is the best fit for your research. If you are not sure, read the appropriate sections before making your selection.

☒ Life sciences ☐ Behavioural & social sciences ☐ Ecological, evolutionary & environmental sciences

For a reference copy of the document with all sections, see nature.com/documents/nr-reporting-summary-flat.pdf

# Life sciences study design

All studies must disclose on these points even when the disclosure is negative.

| | |
|---|---|
| Sample size | NOT APPLICABLE |
| Data exclusions | No data were excluded from the analysis |
| Replication | The methodologies used are reproducible |
| Randomization | Samples were randomly allocated |
| Blinding | Blinding was not applicable to this study |

# Reporting for specific materials, systems and methods

We require information from authors about some types of materials, experimental systems and methods used in many studies. Here, indicate whether each material, system or method listed is relevant to your study. If you are not sure if a list item applies to your research, read the appropriate section before selecting a response.

## Materials & experimental systems

| n/a | Involved in the study | |
|---|---|---|
| ☐ | ☒ | Antibodies |
| ☒ | ☐ | Eukaryotic cell lines |
| ☒ | ☐ | Palaeontology and archaeology |
| ☐ | ☒ | Animals and other organisms |
| ☒ | ☐ | Clinical data |
| ☒ | ☐ | Dual use research of concern |

## Methods

| n/a | Involved in the study | |
|---|---|---|
| ☒ | ☐ | ChIP-seq |
| ☒ | ☐ | Flow cytometry |
| ☒ | ☐ | MRI-based neuroimaging |

## Antibodies

| | |
|---|---|
| Antibodies used | Plates pre-coated with antibodies specific for the targets were used for quantification of LTA and CRP |
| Validation | pre-coated plates were provided in the MyBioSource (LTA quantization) and Invitrogen (CRP quantization) quantization ELISA kits |

# Animals and other research organisms

Policy information about studies involving animals; ARRIVE guidelines recommended for reporting animal research, and Sex and Gender in Research

| | |
|---|---|
| Laboratory animals | Mus musculus CD1 female mice (Envigo) |
| Wild animals | The animals were sacrificed at the end of the experiment by CO2.<br>The study did not involve wild animals. |
| Reporting on sex | Only female mice were used for better coexistence compatibility. |
| Field-collected samples | The samples collected were immediately processed. |
| Ethics oversight | All procedures involving animals were approved by the Italian Ministry of Health, 14 January 2016, Protocol 34/2016-PR. |

Note that full information on the approval of the study protocol must also be provided in the manuscript.
